# Molecular identification of Leishmania infection in the most relevant sand fly species and in patient skin samples from a cutaneous leishmaniasis focus, in Morocco

**DOI:** 10.1371/journal.pntd.0006315

**Published:** 2018-03-02

**Authors:** Idris Mhaidi, Sofia El Kacem, Mouad Ait Kbaich, Adil El Hamouchi, M’hammed Sarih, Khadija Akarid, Meryem Lemrani

**Affiliations:** 1 Laboratory of Parasitology and Vector-Borne-Diseases, Institut Pasteur du Maroc, Casablanca, Morocco; 2 Molecular Genetics and Immunophysiopathology Research Team, Health and Environment Laboratory, Hassan II University of Casablanca, Aïn Chock Faculty of Sciences, Morocco; 3 Laboratory of Biology and Health, Faculty of Sciences Ben M'Sik, Hassan II University, Casablanca, Morocco; National Institutes of Health, UNITED STATES

## Abstract

**Background:**

Cutaneous leishmaniasis (CL) is an infectious disease caused by various species of *Leishmania* and transmitted by several species of sand flies. CL is among the most neglected tropical diseases, and it has represented a major health threat over the past 20 years in Morocco. The main objectives of this study were to identify relevant sand fly species and detect *Leishmania* infection in the most prevalent species and patient skin samples in Taza, a focus of CL in North-eastern Morocco.

**Methodology and finding:**

A total of 3672 sand flies were collected by CDC miniature light traps. Morphological identification permitted the identification of 13 species, namely 10 *Phlebotomus* species and 3 *Sergentomyia* species. *P*. *longicuspis* was the most abundant species, comprising 64.08% of the total collected sand flies, followed by *P*. *sergenti* (20.1%) and *P*. *perniciosus* (8.45%). Using nested-kDNA PCR, seven pools of *P*. *sergenti* were positive to *Leishmania tropica* DNA, whereas 23 pools of *P*. *longicuspis* and 4 pools of *P*. *perniciosus* tested positive for *Leishmania infantum* DNA. The rates of *P*. *longicuspis* and *P*. *perniciosus Leishmania* infection were 2.51% (23/915) and 7.27% (4/55), respectively, whereas the infection prevalence of *P*. *sergenti was* 3.24%. We also extracted DNA from lesion smears of 12 patients suspected of CL, among them nine patients were positive with enzymatic digestion of ITS1 by *Hae*III revealing two profiles. The most abundant profile, present in eight patients, was identical to *L*. *infantum*, whereas *L*. *tropica* was found in one patient. The results of RFLP were confirmed by sequencing of the ITS1 DNA region.

**Conclusion:**

This is the first molecular detection of *L*. *tropica* and *L*. *infantum* in *P*. *sergenti* and *P*. *longicuspis*, respectively, in this CL focus. Infection of *P*. *perniciosus* by *L*. *infantum* was identified for the first time in Morocco. This study also underlined the predominance of *L*. *infantum* and its vector in this region, in which *L*. *tropica* has been considered the causative agent of CL for more than 20 years.

## Author summary

Two types of leishmaniasis are endemic in Morocco: visceral leishmaniasis (VL) and cutaneous leishmaniasis (CL). VL is caused by *Leishmania infantum*, a species that is responsible for sporadic cutaneous leishmaniasis, in addition to *L*. *major* and *L*. *tropica*, which has the largest geographic distribution in Morocco. Taza province is considered as an area of mixed focus wherein VL and CL coexist. However, since the mid-1990s, this region is particularly focused upon for CL due to the presence of *L*. *tropica*. Recently, an *L*. *infantum* dermotropic variant has also been detected in CL patients. This situation calls for an in depth epidemiological investigation to identify with certainty the vectors of both *L*. *infantum* and *L*. *tropica* in this area. Our results highlight the dominance of *L*. *infantum* in cutaneous leishmaniasis patients, this species seems to invade *L*. *tropica*'s field. Moreover, two phlebotomines species have been incriminated in the transmission of *L*. *infantum*, whereas the *L*. *tropica*’s vector has been confirmed. Thus, in light of our findings, the health authorities should implement control measures to counter the spread of both *L*. *infantum* and *L*. *tropica* in Taza province.

## Introduction

Leishmaniasis comprises a group of diseases that are caused by various intracellular protozoa species of the genus *Leishmania* and transmitted by sand flies (Diptera, Phlebotominae). Leishmaniasis is responsible for considerable rates of morbidity and mortality globally, and it affects Mediterranean and other endemic countries, putting a population of 350 million people at risk of infection. The overall prevalence of leishmaniasis is estimated as 12 million cases worldwide, and the global yearly incidence of all clinical forms of the disease is 1.3 million [1] In the Mediterranean basin, two clinico-epidemiological forms of leishmaniasis are endemic: visceral leishmaniasis (VL) and cutaneous leishmaniasis (CL) [2].

VL is a zoonotic disease caused by *Leishmania infantum* infection. The disease tends to be relatively chronic, and children are especially affected [3]. Dogs represent the principal reservoir host in the entire Mediterranean basin [4,5]. CL is often described as a group of diseases because of the varied spectrum of clinical manifestations, which range from small cutaneous nodules to gross mucosal tissue destruction. CL can be caused by several *Leishmania* spp. Despite its increasing worldwide incidence, it is rarely fatal, and thus, CL has become a neglected disease.

In Morocco, CL constitutes a major health threat consisting of three nosogeographic entities: anthroponotic cutaneous leishmaniasis (ACL) caused by *Leishmania tropica* and transmitted by *Phlebotomus* (*Paraphlebotomus*) *sergenti* [6,7]; zoonotic cutaneous leishmaniasis (ZCL) caused by *Leishmania major*, which has been known to exist in the vast area of the arid pre-Saharan region for over a century [8]. *Phlebotomus papatasi* and *Meriones shawi* are the vector and reservoir, respectively, of *L*. *major* [9]; and sporadic CL, which is caused by *L*. *infantum* MON-24 and is present mainly in Northern Morocco, in which ZVL caused by *L*. *infantum* MON-1 is endemic [10–12]. The phlebotomine sand fly vectors of *L*. *infantum* are considered to be *Phlebotomus perniciosus*, *Phlebotomus longicuspis* and *Phlebotomus ariasi* [13]. Whereas the last two species have been already reported to be infected by *L*. *infantum* [4,14], the role of *P*. *perniciosus* as a vector of *L*. *infantum* has never been confirmed in Morocco.

Recently, the epidemiological profile of leishmaniasis in Morocco has changed. Specifically, the predominance of VL in Northern Morocco and CL in Southern Morocco is no longer accurate, as the geographic distribution areas of the three medically important *Leishmania* species overlap. Indeed, *L*. *tropica* has been found in established *L*. *major* foci [15]. In addition, cases of VL have been reported in arid endemic areas of ZCL [16], and the dermotropic variant of *L*. *infantum* has spread to areas considered foci of CL due to the presence of *L*. *tropica* [12,17]. This is the case in Taza province, considered the first focus of CL caused by *L*. *tropica* in Northern Morocco. Indeed, in the mid-1990s, this region experienced a rapid expansion of CL due to *L*. *tropica* MON-102 [18]. Recently reported data identified *L*. *infantum* as the causative agent of CL in 41% of infected patients in this province [17]. Despite the existence of both CL and VL and co-existence of *L*. *tropica* and *L*. *infantum* in this region [17,18], data on the vectors of *Leishmania* transmission is not available.

Therefore, this study sought to detect and identify the *Leishmania* parasite responsible for the recent cases of CL and the putative vector species in Taza city, a mixed focus of CL and VL in Northern Morocco.

## Materials and methods

### Study region

**Fig 1:**
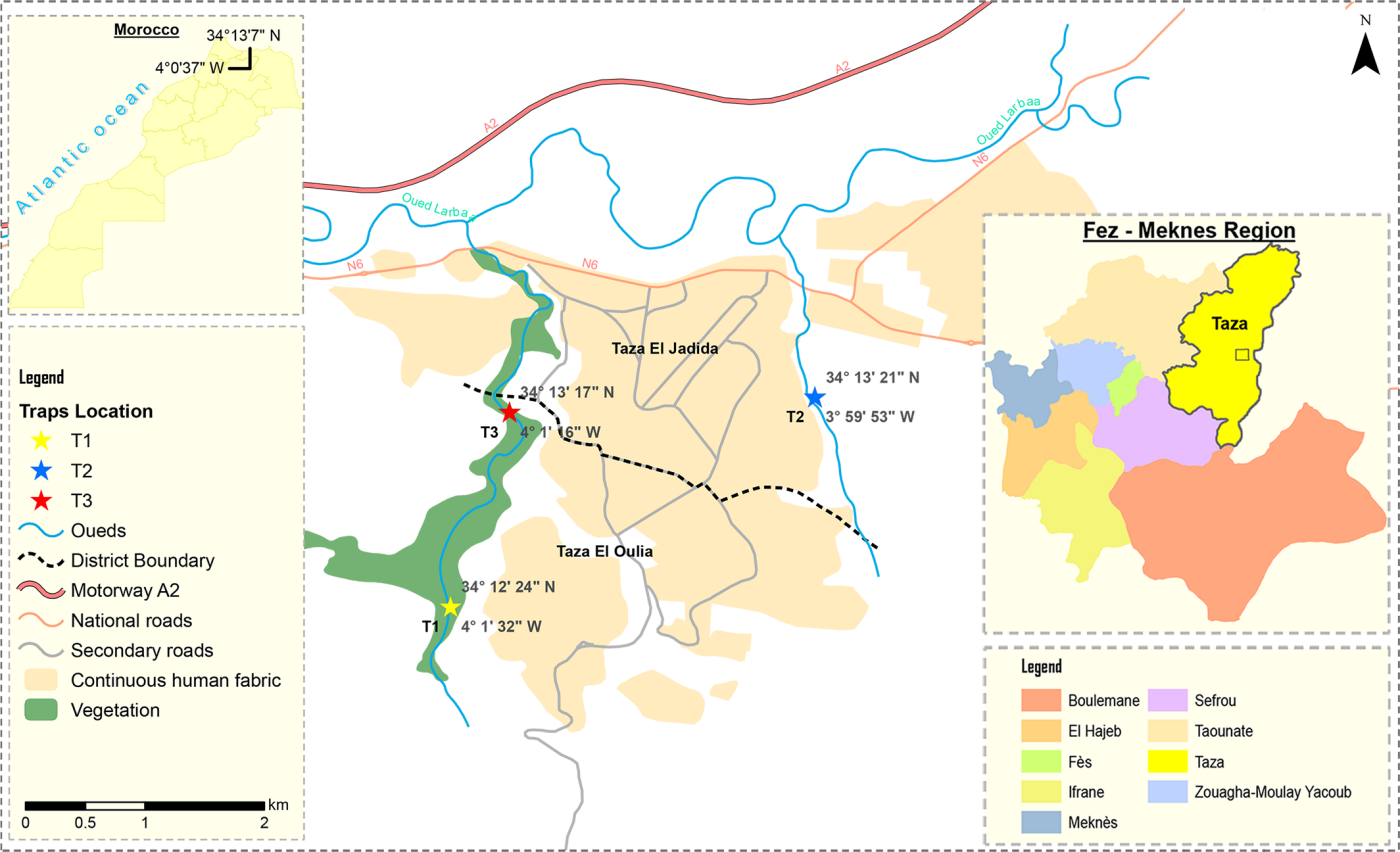
Map of Morocco’s Taza province showing the neighbourhoods sites in which the survey was conducted. The map was created using ArcGIS v10 software. The base layers used to create this figure were obtained from Sentinel-2.

This study was conducted in peri-urban areas of Taza, situated in North-eastern Morocco in the corridor between the Rif and Middle Atlas mountains (Fig 1).

Taza is situation at 550-m elevation. The climate is seasonal, shifting from cool in winter to hot in summer. The temperature varies between 3.2 and 44.5°C. Although annual rainfall in the city has ranged between 84 and 120 mm over the last 8 years, the amount can reach 593 mm per year [19].

### Collection and identification of sand flies

Phlebotomine sand flies were caught monthly on 3 consecutive nights between July and October 2015 in three peri-urban sites (Fig 1), using 3 CDC light traps per site placed inside houses and domestic animal shelters.

The traps were set before sunset and collected at approximately 6 am the next day. The collected sand flies were then placed in 1.5-ml Eppendorf tubes, transferred in dry ice to our laboratory and kept frozen in −80°C for further processing and identification.

Sand fly specimens were washed in sterile distilled water, and the head and genitalia of each sand fly were removed and mounted on microscope slides using the solution of Marc-André (40 g of chloral hydrate, 30 ml glacial acetic acid and 30 ml of distilled water) for morphological identification using the Moroccan morphologic key [20,21]. The remainder of the sand fly body was stored in sterile Eppendorf microtubes for molecular use. To distinguish *P*. *perniciosus* and *P*. *longicuspis*, we used criteria based on the morphological features of their genitalia and the number of coxite hairs [22,23]. For the female subgenus *Larroussius*, they were separated by examining the dilatation of the distal part of spermathecal ducts [24]. In the case of males, the identification was based on the morphological features of the copulatory valve and by the number of coxite hair. *P*. *perniciosus* is characterized by two forms: (i) copulatory valves with a bifid apex; (ii) copulatory valves with a curved apex. The number of coxite hair is not higher than 18. *P*. *longicuspis* has a copulatory valve ending with a single and long point with a mild curve at the edge, and has 21 or more coxite hairs [23,25].

### Human sample collection

Under the National Program of Fight against Leishmaniasis conducted in Taza by regional health authorities, we participated in active screening for 4 days in April 2015. The screening involved schools, nurseries and houses in neighbourhoods in which cases of CL were commonly reported. We also collected clinical samples from patients at health centres.

CL was suspected in 12 patients. Before sampling, we completed a questionnaire regarding information about each patient (code, age, gender, address, history of travel) and the lesions (number, location, onset of the disease, clinical characteristics).

The lesions were cleaned by Alcohol 70% and the tissue samples were collected by dermal scraping from suspected CL patients from the edge of lesions, after which slide smears were prepared, fixed with absolute methanol and stained with Giemsa for microscopic examination. The whole slides were analysed using a ×100 immersion objective.

### DNA extraction from sand flies and slide smears

Total DNA was extracted from slide smears and monospecific pools of sand flies using the phenol chloroform method. Pools of unengorged females of *P*. *longicuspis*, *P*. *perniciosus* and *P*. *sergenti* were homogenised in 200 μl of phosphate-buffered saline (PBS 1×) using a disposable pestle. After centrifugation at 6000 × *g* for 2 min, the supernatants were used for DNA extraction. DNA quantification was determined using a NanoDrop (Thermo Scientific).

### Detection and identification of *Leishmania* species via internal transcribed spacer region (ITS1)-PCR-restriction fragment length polymorphism (RFLP) using human clinical samples

The ITS1 was amplified using primers LITSR and L5.8S following the protocol of Schonian *et al*. [26]. A negative control (without DNA) was used for each PCR run. To identify *Leishmania* species, the positive PCR products of 350 bp in size were subjected to enzymatic restriction by *Hae*III for 2 h at 37°C. RFLPs were analysed by electrophoresis on 3% agarose gel containing ethidium bromide. A 100-bp DNA size marker was used (HyperLadder 100bp Plus). The restriction profiles were compared to reference strains for *L*. *infantum* (MHOM/TN/80/IPT1), *L*. *major* (MHOM/SU/73/5ASKH) and *L*. *tropica* (MHOM/SU/74/K27).

### Molecular detection and identification of *Leishmania* species in unengorged females of *P*. *longicuspis*, *P*. *perniciosus* and *P*. *sergenti* by minicircle kinetoplast DNA (kDNA) nested PCR

The detection and identification of *Leishmania* parasites were performed via nested PCR amplification of kDNA as previously described [27]. Two PCR stages were performed in two separate tubes. The first-stage used the forward primer CSB2XF and reverse primer CSB1XR and the second stage used the nested forward primer 13Z with the nested reverse primer LiR. These primers allowed the amplification of the variable region of kDNA of *Leishmania* species, giving a specific molecular weight for each species as follows: 560 bp for *L*. *major*, 680 bp for *L*. *infantum* and 750 bp for *L*. *tropica* [27,28]. Cross-contamination was monitored using negative controls for sample DNAs and solutions of all PCR reagents used. The amplification products of nested PCR were separated and confirmed by electrophoresis in an ethidium bromide-stained 2% agarose gel.

### Sequencing and phylogenetic analysis

The obtained 350-bp ITS1-PCR products were purified using exonuclease I/shrimp alkaline phosphatase (GE Healthcare, US). The sequencing reaction was performed in both directions using BigDye Terminator version 3.1 (Applied Biosystems, Foster City, CA, USA). The resulting forward and reverse sequences were aligned. A total of nine partial ITS1 DNA sequences, in addition to the studied sequences, were selected from GenBank, including five sequences from *L*. *infantum* (AJ634361, GQ367486, KX664452, AJ634339 and AJ634355), four sequences from *L*. *tropica* (KP202104, KU194923, KX599337 and KP202102) and one sequence from *L*. *major* (AJ000310). Phylogenetic analysis was performed with MEGA version 7 software using the neighbour-joining and Kimura 2-parameter models. The tree topology was supported by 1000 bootstrap replicates.

### Ethics approval and consent to participate

Written informed consent was obtained from all the adults who participated in the study. Consent for inclusion of young children, was obtained from parents or guardians. The study and the protocols were approved by the Ethics Committee for Biomedical Research (CERB) of the Faculty of Medicine and Pharmacy, Rabat, Morocco.

## Results

### Phlebotomine sand fly fauna

A total of 3,583 sand flies (2,485 females and 1,098 males, Table 1) were collected in three peri-urban neighbourhoods of Taza (Fig 1). Our results revealed a monthly evolution that varied among the species. Specifically, the evolution was monophasic for some species and biphasic for others. *P*. *longicuspis*, the most abundant sand fly species, had one peak of activity in August, whereas *P*. *sergenti*, the second most prevalent species, exhibited two peaks of activity in July and September (Table 1).

**Table 1:** Species diversity, abundance (A) and relative frequency (F) of sand fly collected from July to October in Taza city.

	July		August		September		October		Total		
	A	F	A	F	A	F	A	F	A		F
	M+F	(%)	M+F	(%)	M+F	(%)	M+F	(%)	M	F	(%)
***P*. *longicuspis***	78	14.82	1355	77.03	763	71.37	158	85.40	498	1856	65.69
***P*. *sergenti***	364	69.20	151	8.58	238	22.26	10	5.40	400	363	21.29
P. perniciosus*	56	10.64	156	8.86	41	3.83	5	2.40	149	164	8.73
***S*. *minuta***	13	2.47	54	3.06	12	1.12	1	0.54	17	53	1.9
***P*. *papatasi***	9	1.71	15	0.85	6	0.56	-	-	17	13	1.95
***P*. *perfiliewi***	-	-	9	0.51	7	0.65	4	2.16	-	20	0.55
***P*. *bergeroti***	-	-	1	0.05	-	-	6	3.24	-	7	0.19
***P*. *ariasi***	1	0.19	6	0.34	-	-	-	-	-	7	0.19
***P*. *kazeruni***	2	0.38	-	-	-	-	-	-	2	-	0.05
***P*. *langeroni***	-	-	-	-	1	0.09	-	-	1	-	0.02
***P*. *alexandri***	-	-	-	-	1	0.09	-	-	1	-	0.02
***S*. *antennata***	3	0.57	10	0.56	-	-	-	-	13	-	0.36
***S*. *dreyfussi***	-	-	2	0.11	-	-	1	0.54	-	3	0.08
**Total**	589	100	1759	100	1069	100	195	100	1098	2485	

*Including typical (PN) and atypical (PNA) form of *P*. *perniciosus*

Morphological identification revealed the presence of 13 species, including 10 belonging to the genus *Phlebotomus* and 3 in the genus *Sergentomyia* (Table 1). The most prevalent species was *P*. *longicuspis*, with a relative abundance of 64.08% of the total collected sand flies. *P*. *sergenti* and *P*. *perniciosus* were the next most abundant species, comprising 20.78 and 8.52%, respectively of the specimens. These three species constituted 93.11% of the total number of collected sand flies. The remaining 3.55% consisted of *P*. *papatasi*, *P*. *bergeroti*, *P*. *ariasi*, *P*. *dreyfussi*, *P*. *kazeruni*, *P*. *langeroni*, *P*. *alexandri*, *P*. *perfiliewi*, *S*. *minuta* and *S*. *antennata* (Table 1). The sex ratio was 0.43, indicating that more females were collected by CDC light trap in this area.

### *Leishmania*-infected flies and parasite typing by kDNA nested PCR

Unengorged female sand flies of the most prevalent species collected in this study, namely *P*. *longicuspis*, *P*. *sergenti* and *P*. *perniciosus*, were organised in a total of 54 pools, each containing up to 30 sand flies (39 *P*. *longicuspis* pools, 4 *P*. *perniciosus* pools and 11 *P*. *sergenti* pools, Table 2). The pools were screened for *Leishmania* infection using kDNA nested PCR. *Leishmania* DNA was amplified from 23 *P*. *longicuspis* and 4 *P*. *perniciosus* pools, giving a single band of 680 bp corresponding to *L*. *infantum*, as well as seven *P*. *sergenti* pools, giving an expected single band of 750 bp corresponding to *L*. *tropica*. Hence, the overall minimum *L*. *infantum* infection rates based on the positive PCR of *P*. *longicuspis* and *P*. *perniciosus* were 2.51 and 7.27%, respectively. The *L*. *tropica* infection rate within *P*. *sergenti* was evaluated as 3.24% (Table 2).

**Table 2:** Detection and identification of *L*. *infantum* in both *P*. *longicuspis and P*. *perniciosus* and *L*. *tropica* DNA in *P*. *sergenti*.

Subgenus	*Larroussius*						*Paraphlebotomus*		
Species	*P*. *longicuspis*			*P*. *perniciosus*			*P*. *sergenti*		
	Number of Pools	*L*. *infantum* infection	Rate of infection	Number of Pools	*L*. *infantum* infection	Rate of infection	Number of Pools	*L*. *tropica* infection	Rate of infection
July	3	1	1/44 (2.27%)	1	1	1/12 (8.33%)	4	3	3/76 (3.94%)
August	17	11	11/394 (2.79%)	2	2	2/28 (7.14%)	3	2	2/59 (3.38%)
September	15	7	7/397(1.76%)	1	1	1/15 (6.66%)	4	2	2/81 (5.26%)
October	4	4	4/80 (5%)	-	-	-	-	-	-
Total	39	23	23/915 (2.51%)	4	4	4/55 (7.27%)	11	7	7/216 (3.24%)

### *Leishmania*-infected patients and parasite typing by ITS1-PCR-RFLP

Among the 12 patients suspected to have CL based on clinical criteria, seven were found to be positive on microscopic examination. ITS1-PCR was more sensitive, as it identified nine CL-positive patients. Enzymatic digestion by *Hae*III revealed two profiles. The first consisted of two bands (185 and 57/53 bp), as observed for *L*. *tropica* (MHOM/SU/74/K27), and the second profile (184, 72 and 55 bp) was the most abundant band pattern; identical to *L*. *infantum* MHOM/FR/78/LEM75 and detected in eight patients. As for *L*. *tropica* it was observed in only one patient (Table 3).

**Table 3:** ITS1-PCR-RFLP results of CL patients.

N° of patient	gender	PCR-ITS1	RFLP-RsaI
1	M	Positif	*L*. *infantum*
2	F	Negatif	*-*
3	M	Negatif	*-*
4	M	Positif	*L*.*infantum*
5	F	Positif	*L*. *infantum*
6	F	Positif	*L*. *infantum*
7	M	Positif	*L*. *infantum*
8	M	Negatif	*-*
9	F	Positif	*L*. *infantum*
10	F	Positif	*L*. *infantum*
11	F	Positif	*L*. *tropica*
12	M	Positif	*L*. *infantum*

### *Leishmania* DNA sequencing and phylogenetic analysis

To confirm the PCR-RFLP identification results, the ITS1-PCR products were sequenced and subjected to NCBI-BLAST analysis for homology (http://blast.ncbi.nlm.nih.gov). The ITS1 sequences exhibited a length range of 300–350 bp. The identity of all isolates from patients with CL was in agreement with the ITS1-PCR-RFLP results, indicating that the sequences KY973658, KY973656 and KY973661 were similar (97%) to the Spanish *L*. *infantum* sequence deposited in GenBank under accession number AJ634355, whereas KY973656, KY973657 and KY973662, were similar (98%) to the Moroccan *L*. *infantum* sequence deposited in GenBank under accession number KX664452. Conversely, the sequence KY974310 was similar to the Moroccan *L*. *tropica* sequence deposited in GenBank under accession number KP202104 (99%).

**Fig 2:**
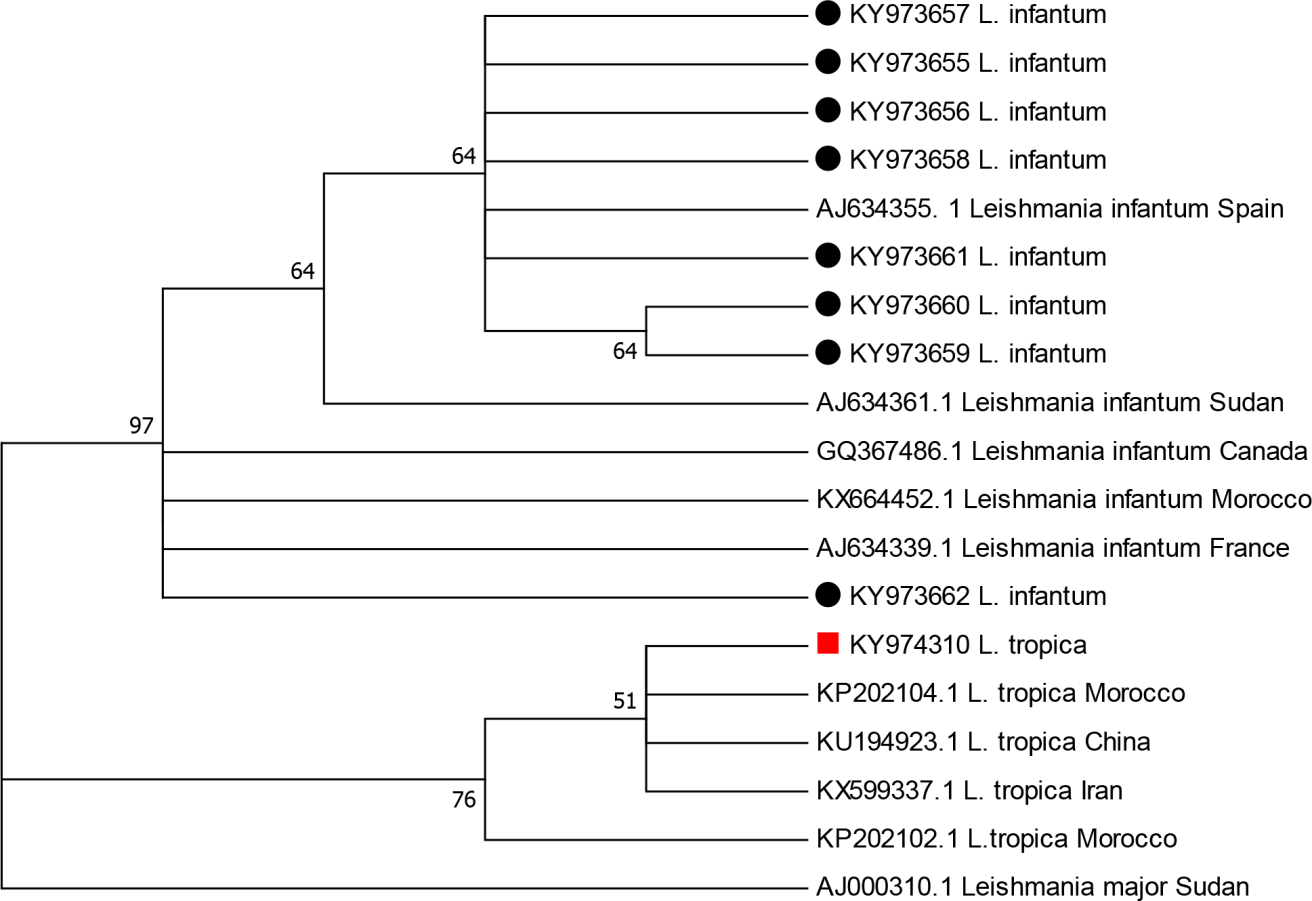
Maximum likelihood phylogenetic tree based on *Leishmania* ITS1 sequences showing the relationships of the sequences of *L*. *tropica* and *L*. *infantum*.

The phylogenetic analysis based on ITS1 sequences generated in this work and other sequences from NCBI revealed that all *L*. *infantum* isolates are clustered together, whereas *L*. *tropica* and *L*. *major* each appear in different clusters. The obtained tree also revealed a level of polymorphism among the *L*. *infantum* Moroccan isolates (four distinct genotypes were identified among eight isolates, Fig 2).

## Discussion

Despite the increasing annual prevalence of leishmaniasis in Taza, little is known regarding the epidemiological aspects of VL and CL in this area. Species identification of the vectors of leishmaniasis and etiological agent typing in these vectors represent steps forward in understanding the epidemiology of leishmaniasis, which will permit the implementation of improved disease control programmes. The present study aimed to clarify the diversity and frequency of sand flies to identify the *Leishmania* species infecting medically important sand flies as well as patients with suspected CL. The focus of Taza is of particular importance due to the co-existence of VL and CL [17]. Whereas the visceral form is caused only by *L*. *infantum*, CL is caused by both *L*. *infantum* and *L*. *tropica*. However, there are no data on vector incrimination. The only global entomological survey focusing on the diversity and frequency of sand flies in this province was performed two decades ago. Nine sand fly species were identified, *P*. *sergenti* was the dominant species comprising 43% of the total phlebotomine species collected, following by *P*. *longicuspis* (19.5%). Throughout the study period, the activity of sand flies was marked by the dominance of *P*. *longicuspis* with one peak in August, followed by *P*. *sergenti*, which was abundant in July and declined in October. Such a pattern of seasonal abundance of *P*. *sergenti* was observed in Chichaoua, where an entomological survey was performed during July 2002 to December 2003, the peak of density of *P*. *sergenti* was reached during July and August, then the density decreased from September through November [29]. The activity period of Mediterranean adult sand flies is typically seasonal [30]. It was reported that the abundance of phlebotomine sand flies varies between species and site of collection; seasonal and monthly density patterns differ also between years, as the abundance of sand fly is impacted by the annual climate variation and local weather event [31].

In Morocco, the presence of *P*. *longicuspis* was reported in different areas, ranging from the sub-humid belt to the Sahara. The geographical distribution area of this species is characterised by an intermediate altitude, Saharan climate and sandy loam soil texture [32,33]. This species appears to have a similar distribution in Tunisia, in which it has been found in all bio-geographical areas including the Saharan bio-climatic zone, where its relative abundance reaches 60% [34].

The distribution of species belonging to the subgenus *Larroussius* has been widely studied [22,35]; however, little is known about their vectorial role in Morocco. *P*. *longicuspis* was considered the vector of *L*. *infantum*, as it was the only representative species of *Larroussius* subgenus in a ZVL focus in the arid zone in Morocco [16]. Similarly, *P*. *longicuspis* has been incriminated as a vector of *L*. *infantum* in a VL focus in Northern Morocco because it had sufficient abundance for transmitting *L*. *infantum* [4]. However, Es-sette *et al*. (2014) reported for the first time the natural infection of *P*. *longicuspis* by *L*. *infantum* DNA and its anthropophilic character in a CL focus in Northern Morocco [36]. In Algeria, *P*. *longicuspis* is considered a vector of *L*. *infantum* in an endemic VL focus, in which this phlebotomine species has been found to be infected by *L*.*infantum* DNA [37]. The role of *P*. *longicuspis* in *L*. *infantum* transmission was also proved by Rioux and Lanotte in a CL focus [38]. Our findings support the role of *P*. *longicuspis* in *L*. *infantum* transmission in this mixed focus in which both CL and VL are caused by *L*. *infantum* [17]. Indeed, the high abundance of *P*. *longicuspis* and its natural infection by *L*. *infantum* provide further evidence of its role as vector of *L*. *infantum*, especially considering its vector competence in *L*. *infantum* transmission has been widely demonstrated [36,39,40].

*P*. *perniciosus* is considered the principal vector of *L*. *infantum* in the Western Mediterranean basin, especially in Tunisia, Algeria, Spain and Italy [41–44]. It has also been found in all bio-geographical areas of Algeria, Tunisia and Morocco [22]. This species is more common in wet floors, sub-humid areas and semi-arid high altitudes [35]. In this study, we reported for the first time the natural infection of *P*. *perniciosus* by *L*. *infantum* DNA in the country. The infection rate was estimated as 7.27% even though the abundance of *P*. *perniciosus* did not exceed 8.52% of the collected species. The infection rate varies between foci, and it is dependent on the techniques used for *Leishmania* detection. Indeed, in South-western Madrid, 58.5% of *P*. *perniciosus* specimens were positive for *L*. *infantum* infection using kDNA PCR methods [45], whereas in Northern and Central Tunisia, the prevalence of *L*. *infantum* infection within *P*. *perniciosus* was 0.16% according to ITS-rDNA gene nested PCR [39].

CL due to *L*. *tropica* has been identified in Taza province since 1997 [18], as well as neighbouring provinces such as Sefrou [46], Moulay Yacoub [47] and Taounate [4]. It is transmitted through *P*. *sergenti*, the proven vector of *L*. *tropica* in Morocco [6,48]. This vector was identified in Taza province in 1995, as the prevalent species, it represented 43% of the total capture, followed by *P*. *longicuspis* and *P*. *perniciosus* [18]. Twenty years later, the distribution of sand fly species is completely different; indeed, *P*. *longicuspis* is the most abundant species, comprising 64% of specimens collected in this study, followed by *P*. *sergenti*. The change in the distribution and abundance of *Leishmania* vectors observed in this site is suggested possibly due to long-term climate change. Indeed the global warming was reported to have strong effects on the ecology of *Leishmania* vectors by altering their distribution and influencing their survival and population sizes which affect the epidemiology of leishmaniasis [2,49]. Other studies in Morocco confirmed that the combined effect of climate and environmental factors (night-time temperature, soil water stress, Normalized Difference Vegetation Index and aridity) determine the vector distribution [50]. More in-depth investigation with interdisciplinary approach need to be done to explain the change of species composition by long-term climate change and to implement a more targeted approach for vector control in the region.

*P*. *sergenti* is the confirmed vector of *L*. *tropica* in North Africa, the Middle East and Central Asia [6,48,51,52]. Screening for *Leishmania* infection within *P*. *sergenti* revealed an infection rate of 3.24% and confirmed the role of *P*. *sergenti* as the vector of *L*. *tropica* in Taza city. This infection rate may be a consequence of the important circulation of *L*. *tropica* in this focus, as the first outbreak of CL caused by *L*. *tropica* occurred in 1995 [18] and this form of CL continues to persist as reported by Hakkour *et al*. [17]. Moreover, our results identified one patient infected by *L*. *tropica* among people with suspected CL lesions, confirming the persistence of this *Leishmania* species in Taza city. Even though *L*. *infantum* is more prevalent (8 out of 9 patients were infected by *L*. *infantum*); this finding is in line with the increasing abundance of species belonging to the subgenus *Larroussius*.

Our results highlight three findings, including the change in the distribution of the sand fly population. Firstly, *P*. *longicuspis* has become more abundant than *P*. *sergenti*, which was more abundant during the first outbreak of CL in 1995 in this region [18]. Secondly, we identified for the first time in Morocco the natural infection of both *P*. *longicuspis* and *P*. *perniciosus* by *L*. *infantum* DNA in the same region, providing evidence that both species participate in the transmission of *L*. *infantum* in this area. Finally, we confirmed that the pattern of CL epidemiology in Taza province is about to change.

### Conclusion

Based on our entomological and epidemiological findings, we believe that environmental changes may have shifted the sand fly distribution, making the subgenus *Larroussius* most prevalent. This has led to the establishment of a stable transmission cycle of *L*. *infantum* and subsequently to the emergence of CL due to *L*. *infantum*, which is invading the area of *L*.*tropica*. In fact, *L*. *infantum* is becoming a problem in Morocco because of its rapid spread throughout the country. Indeed, this *Leishmania* species was reported in several foci previously only considered *L*. *tropica* CL foci. *L*. *infantum* was also identified in *L*. *major* CL foci. Thus, more investigations and control measures are needed in Taza city in light of our findings to counter the spread of both *L*. *infantum* and *L*. *tropica*.
